# Supplementary material for: Pyruvate Dehydrogenase Kinase 1 inhibition mediated oxidative phosphorylation enhancement in cartilage promotes osteoarthritis progression
Source: BMC Musculoskelet Disord. 2023 Jul 20;24:597. doi: 10.1186/s12891-023-06585-6 (PMC10357736; doi:10.1186/s12891-023-06585-6)
Supplement: Supplementary file 1 — Additional file 1. [file 12891_2023_6585_MOESM1_ESM.zip › MF.Gsea.1653627316242/GOMF_METAL_CLUSTER_BINDING.html]

Details for gene set GOMF\_METAL\_CLUSTER\_BINDING[GSEA]

|  || Dataset | 55457\_collapsed\_to\_symbols.cls.cls #OA\_versus\_normal.cls.cls #OA\_versus\_normal\_repos |
| Phenotype | cls.cls#OA\_versus\_normal\_repos |
| Upregulated in class | OA |
| GeneSet | GOMF\_METAL\_CLUSTER\_BINDING |
| Enrichment Score (ES) | 0.3199066 |
| Normalized Enrichment Score (NES) | 1.329654 |
| Nominal p-value | 0.07631579 |
| FDR q-value | 0.38344306 |
| FWER p-Value | 1.0 |
Table: GSEA Results Summary

  

Fig 1: Enrichment plot: GOMF\_METAL\_CLUSTER\_BINDING      
 Profile of the Running ES Score & Positions of GeneSet Members on the Rank Ordered List

  

| SYMBOL | TITLE | RANK IN GENE LIST | RANK METRIC SCORE | RUNNING ES | CORE ENRICHMENT || 1 | GLRX5 | glutaredoxin 5 [Source:HGNC Symbol;Acc:HGNC:20134] | 263 | 0.778 | 0.0233 | Yes |
| 2 | ACO2 | aconitase 2 [Source:HGNC Symbol;Acc:HGNC:118] | 290 | 0.756 | 0.0639 | Yes |
| 3 | FECH | ferrochelatase [Source:HGNC Symbol;Acc:HGNC:3647] | 396 | 0.707 | 0.0955 | Yes |
| 4 | TYW1 | tRNA-yW synthesizing protein 1 homolog [Source:HGNC Symbol;Acc:HGNC:25598] | 523 | 0.650 | 0.1223 | Yes |
| 5 | POLD1 | "DNA polymerase delta 1, catalytic subunit [Source:HGNC Symbol;Acc:HGNC:9175]" | 811 | 0.566 | 0.1319 | Yes |
| 6 | NDUFS7 | NADH:ubiquinone oxidoreductase core subunit S7 [Source:HGNC Symbol;Acc:HGNC:7714] | 1071 | 0.501 | 0.1399 | Yes |
| 7 | FDX1 | ferredoxin 1 [Source:HGNC Symbol;Acc:HGNC:3638] | 1168 | 0.483 | 0.1596 | Yes |
| 8 | NDUFS8 | NADH:ubiquinone oxidoreductase core subunit S8 [Source:HGNC Symbol;Acc:HGNC:7715] | 1208 | 0.474 | 0.1833 | Yes |
| 9 | NDUFS1 | NADH:ubiquinone oxidoreductase core subunit S1 [Source:HGNC Symbol;Acc:HGNC:7707] | 1229 | 0.471 | 0.2082 | Yes |
| 10 | ISCU | iron-sulfur cluster assembly enzyme [Source:HGNC Symbol;Acc:HGNC:29882] | 1530 | 0.423 | 0.2087 | Yes |
| 11 | NDUFV2 | NADH:ubiquinone oxidoreductase core subunit V2 [Source:HGNC Symbol;Acc:HGNC:7717] | 1642 | 0.407 | 0.2230 | Yes |
| 12 | NDUFV1 | NADH:ubiquinone oxidoreductase core subunit V1 [Source:HGNC Symbol;Acc:HGNC:7716] | 1663 | 0.404 | 0.2442 | Yes |
| 13 | NFS1 | NFS1 cysteine desulfurase [Source:HGNC Symbol;Acc:HGNC:15910] | 1741 | 0.393 | 0.2603 | Yes |
| 14 | SDHB | succinate dehydrogenase complex iron sulfur subunit B [Source:HGNC Symbol;Acc:HGNC:10681] | 1818 | 0.382 | 0.2759 | Yes |
| 15 | IREB2 | iron responsive element binding protein 2 [Source:HGNC Symbol;Acc:HGNC:6115] | 2215 | 0.337 | 0.2640 | Yes |
| 16 | NUBP1 | nucleotide binding protein 1 [Source:HGNC Symbol;Acc:HGNC:8041] | 2568 | 0.295 | 0.2532 | Yes |
| 17 | NUBPL | nucleotide binding protein like [Source:HGNC Symbol;Acc:HGNC:20278] | 2569 | 0.295 | 0.2698 | Yes |
| 18 | RSAD2 | radical S-adenosyl methionine domain containing 2 [Source:HGNC Symbol;Acc:HGNC:30908] | 2619 | 0.288 | 0.2822 | Yes |
| 19 | LIAS | lipoic acid synthetase [Source:HGNC Symbol;Acc:HGNC:16429] | 2917 | 0.259 | 0.2737 | Yes |
| 20 | ELP3 | elongator acetyltransferase complex subunit 3 [Source:HGNC Symbol;Acc:HGNC:20696] | 2974 | 0.253 | 0.2835 | Yes |
| 21 | NDUFS2 | NADH:ubiquinone oxidoreductase core subunit S2 [Source:HGNC Symbol;Acc:HGNC:7708] | 3072 | 0.243 | 0.2897 | Yes |
| 22 | NFU1 | NFU1 iron-sulfur cluster scaffold [Source:HGNC Symbol;Acc:HGNC:16287] | 3086 | 0.241 | 0.3022 | Yes |
| 23 | NTHL1 | nth like DNA glycosylase 1 [Source:HGNC Symbol;Acc:HGNC:8028] | 3219 | 0.228 | 0.3048 | Yes |
| 24 | GLRX3 | glutaredoxin 3 [Source:HGNC Symbol;Acc:HGNC:15987] | 3336 | 0.218 | 0.3080 | Yes |
| 25 | EXO5 | exonuclease 5 [Source:HGNC Symbol;Acc:HGNC:26115] | 3487 | 0.204 | 0.3078 | Yes |
| 26 | ERCC2 | "ERCC excision repair 2, TFIIH core complex helicase subunit [Source:HGNC Symbol;Acc:HGNC:3434]" | 3544 | 0.198 | 0.3146 | Yes |
| 27 | CIAO3 | cytosolic iron-sulfur assembly component 3 [Source:HGNC Symbol;Acc:HGNC:14179] | 3784 | 0.178 | 0.3061 | Yes |
| 28 | DPYD | dihydropyrimidine dehydrogenase [Source:HGNC Symbol;Acc:HGNC:3012] | 3830 | 0.173 | 0.3123 | Yes |
| 29 | BOLA2 | bolA family member 2 [Source:HGNC Symbol;Acc:HGNC:29488] | 4010 | 0.155 | 0.3071 | Yes |
| 30 | CISD1 | CDGSH iron sulfur domain 1 [Source:HGNC Symbol;Acc:HGNC:30880] | 4104 | 0.146 | 0.3081 | Yes |
| 31 | ISCA1 | iron-sulfur cluster assembly 1 [Source:HGNC Symbol;Acc:HGNC:28660] | 4116 | 0.144 | 0.3154 | Yes |
| 32 | CIAPIN1 | cytokine induced apoptosis inhibitor 1 [Source:HGNC Symbol;Acc:HGNC:28050] | 4161 | 0.141 | 0.3199 | Yes |
| 33 | PRIM2 | DNA primase subunit 2 [Source:HGNC Symbol;Acc:HGNC:9370] | 4491 | 0.114 | 0.3007 | No |
| 34 | MUTYH | mutY DNA glycosylase [Source:HGNC Symbol;Acc:HGNC:7527] | 5252 | 0.052 | 0.2444 | No |
| 35 | UQCRFS1 | "ubiquinol-cytochrome c reductase, Rieske iron-sulfur polypeptide 1 [Source:HGNC Symbol;Acc:HGNC:12587]" | 5509 | 0.032 | 0.2263 | No |
| 36 | BRIP1 | BRCA1 interacting helicase 1 [Source:HGNC Symbol;Acc:HGNC:20473] | 5885 | 0.003 | 0.1973 | No |
| 37 | GLRX2 | glutaredoxin 2 [Source:HGNC Symbol;Acc:HGNC:16065] | 6116 | -0.014 | 0.1801 | No |
| 38 | AOX1 | aldehyde oxidase 1 [Source:HGNC Symbol;Acc:HGNC:553] | 6243 | -0.024 | 0.1716 | No |
| 39 | ABAT | 4-aminobutyrate aminotransferase [Source:HGNC Symbol;Acc:HGNC:23] | 6804 | -0.063 | 0.1316 | No |
| 40 | RSAD1 | radical S-adenosyl methionine domain containing 1 [Source:HGNC Symbol;Acc:HGNC:25634] | 7355 | -0.106 | 0.0948 | No |
| 41 | KIF4A | kinesin family member 4A [Source:HGNC Symbol;Acc:HGNC:13339] | 7480 | -0.117 | 0.0917 | No |
| 42 | MOCS1 | molybdenum cofactor synthesis 1 [Source:HGNC Symbol;Acc:HGNC:7190] | 7714 | -0.135 | 0.0811 | No |
| 43 | CDKAL1 | CDK5 regulatory subunit associated protein 1 like 1 [Source:HGNC Symbol;Acc:HGNC:21050] | 7761 | -0.138 | 0.0853 | No |
| 44 | FXN | frataxin [Source:HGNC Symbol;Acc:HGNC:3951] | 8057 | -0.160 | 0.0714 | No |
| 45 | XDH | xanthine dehydrogenase [Source:HGNC Symbol;Acc:HGNC:12805] | 8366 | -0.183 | 0.0577 | No |
| 46 | ACO1 | aconitase 1 [Source:HGNC Symbol;Acc:HGNC:117] | 9426 | -0.266 | -0.0097 | No |
| 47 | ABCE1 | ATP binding cassette subfamily E member 1 [Source:HGNC Symbol;Acc:HGNC:69] | 9675 | -0.289 | -0.0128 | No |
| 48 | ETFDH | electron transfer flavoprotein dehydrogenase [Source:HGNC Symbol;Acc:HGNC:3483] | 10203 | -0.341 | -0.0346 | No |
| 49 | CDK5RAP1 | CDK5 regulatory subunit associated protein 1 [Source:HGNC Symbol;Acc:HGNC:15880] | 10870 | -0.413 | -0.0632 | No |
| 50 | POLE | "DNA polymerase epsilon, catalytic subunit [Source:HGNC Symbol;Acc:HGNC:9177]" | 11026 | -0.433 | -0.0509 | No |
| 51 | RPS3 | ribosomal protein S3 [Source:HGNC Symbol;Acc:HGNC:10420] | 11245 | -0.463 | -0.0418 | No |
| 52 | REV3L | "REV3 like, DNA directed polymerase zeta catalytic subunit [Source:HGNC Symbol;Acc:HGNC:9968]" | 11469 | -0.498 | -0.0311 | No |
| 53 | PPAT | phosphoribosyl pyrophosphate amidotransferase [Source:HGNC Symbol;Acc:HGNC:9238] | 11534 | -0.505 | -0.0076 | No |
| 54 | RTEL1 | regulator of telomere elongation helicase 1 [Source:HGNC Symbol;Acc:HGNC:15888] | 11968 | -0.587 | -0.0083 | No |
| 55 | DNA2 | DNA replication helicase/nuclease 2 [Source:HGNC Symbol;Acc:HGNC:2939] | 12148 | -0.631 | 0.0133 | No |
| 56 | DDX11 | DEAD/H-box helicase 11 [Source:HGNC Symbol;Acc:HGNC:2736] | 12607 | -0.803 | 0.0228 | No |
Table: GSEA details [plain text format]

  

Fig 2: GOMF\_METAL\_CLUSTER\_BINDING      
 Blue-Pink O' Gram in the Space of the Analyzed GeneSet

  

Fig 3: GOMF\_METAL\_CLUSTER\_BINDING: Random ES distribution      
 Gene set null distribution of ES for **GOMF\_METAL\_CLUSTER\_BINDING**
